# Sublingual sufentanil for patient-controlled analgesia during labor induction for pregnancy termination: an effective and well-tolerated approach

**DOI:** 10.1186/s44158-024-00177-z

**Published:** 2024-07-08

**Authors:** Giulia Fierro, Barbara Milan, Elena Buelli, Dario Bugada, Erika Casarotta, Francesco Rizzo, Laura Ongaro, Paolo Gritti, Fabio Previdi, Ferdinando Luca Lorini

**Affiliations:** 1 Department of Emergency and Intensive Care, ASST Papa Giovanni XXIII, P.Za OMS, Bergamo, 24127 Italy; 2 Department of Biomedical Sciences and Public Health, Università Politecnica Delle Marche, Via Tronto 10/a, Ancona, 60020 Italy; 3 Department of Management, Information and Production Engineering, University of Bergamo, Viale G. Marconi 5, Dalmine, BG 24044 Italy

**Keywords:** Medical abortion, Intrauterine fetal death, Labor pain, Sublingual sufentanil, Labor analgesia

## Abstract

**Background:**

Effective pain management during labor induction for pregnancy termination is essential. However, to date, no effective treatment has been identified. The primary aim of this study was to measure the analgesic efficacy of a sufentanil sublingual tablet system during pregnancy termination and patient satisfaction by comparing nulliparous and multiparous women. The secondary aims were to characterize the safety profile by reporting any side effects or adverse events and to determine the need for rescue therapy.

**Methods:**

We conducted an observational, retrospective, single-center study involving 48 women. The data retrieved for analysis included the total and hourly doses of sublingual sufentanil, evaluations of pain management satisfaction using a 5-point rating scale (ranging from 1, indicating “not satisfied” to 5, denoting “completely satisfied”), occurrence of side effects and adverse events, and the rate of rescue analgesic use. Categorical and numerical variables were compared between the two groups, and a correlation analysis was performed.

**Results:**

The median total dose of sufentanil required was 60 mcg. Nulliparous women required a higher dose of sufentanil compared with multiparous women (105 mcg vs. 45 mcg; *P* = 0.01). Additionally, they underwent a longer labor, indirectly measured by the time of device usage (625 min vs. 165 min; *P* = 0.05). Regarding satisfaction, 40 patients (83.4%) were satisfied or completely satisfied, whereas only 8 patients (16.6%) reported dissatisfaction. Multiparous women exhibited higher satisfaction levels than did nulliparous women (*P* = 0.03). No adverse events were reported, and the most common side effects were nausea and vomiting (31.2%). Four patients (12%) required acetaminophen due to insufficient analgesia, with only one patient necessitating a switch to intravenous morphine.

**Conclusions:**

Sublingual sufentanil was effective in both nulliparous and multiparous women with minimal side effects. Therefore, sublingual sufentanil can be considered a valid strategy for analgesia during labor induction for pregnancy termination.

**Supplementary Information:**

The online version contains supplementary material available at 10.1186/s44158-024-00177-z.

## Background

Labor induction (LI) for the termination of pregnancy (TOP) is required in cases of medical abortion or intrauterine fetal death. However, no data are available on the overall incidence of LI in TOP worldwide. Nonetheless, in such circumstances, mothers and families face the risk of severe and prolonged psychological reactions, including post-traumatic stress disorder, emphasizing the necessity for optimal support from all health professionals involved [1–3].

A crucial aspect of labor in TOP is the pain resulting from uterine smooth muscle contractions and the passage of the fetus through the cervix [4]. Predictors of severe pain during TOP include later gestational age, young age, nulliparity, anxiety, depression, and a medical history of dysmenorrhea [5–8].

The International Federation of Gynecology and Obstetrics, Royal College of Obstetricians and Gynecologists, World Health Organization, and the American College of Obstetricians and Gynecologists all advocate for appropriate pain control during LI for TOP [8]. Despite these recommendations, the use of analgesia for TOP remains unexplored. Currently, an optimal analgesic treatment for TOP to reduce pain during LI has not been established [9–11].

According to current evidence, acetaminophen or non-steroidal anti-inflammatory drugs (NSAIDs) combined with scheduled doses of parenteral opioids are beneficial [4, 8]. Studies have shown that patient-controlled analgesia (PCA) leads to greater patient satisfaction and is more successful than non-PCA injections are in controlling pain [12]. Self-administered morphine via PCA has been shown to be superior to nurse-based or continuous infusions. However, the limited availability of PCA pumps, safety issues related to pump preparation, and programming, as well as challenges with low patient compliance — including difficulties with pump management and movement restrictions — render this route of administration infeasible or inaccessible for universal use [13, 14]. In addition, the pharmacokinetics of morphine necessitates time for titrating analgesia, which frequently requires the presence of a doctor for administration.

Recently, the sufentanil sublingual tablet system (SSTS) was approved for clinical use in Europe [15]. The SSTS is a noninvasive, on-demand opioid delivery system. It enables patients to self-administer a fixed dose of 15 mcg of sublingual sufentanil via nanotablets [16]. The SSTS is also characterized by a lockout interval of 20 min which cannot be overridden, thereby reducing the risk of overdose.

Sufentanil, a potent synthetic opioid, is routinely administered with epidural analgesia to control acute pain during labor. It works synergistically as a pure agonist with local anesthetics [17]. Administering sublingual sufentanil enables rapid absorption into the systemic circulation, resulting in a faster onset and a higher rate of successful analgesia [18]. In addition, sublingual sufentanil administered with the SSTS has proven effective in controlling postoperative pain in patients undergoing gynecological and urological surgery, showing a rapid onset and increased success rate of analgesia, compared with intravenous morphine-based PCA [19]. Considering these positive results, we hypothesized that the STSS could be extended to obstetric settings to manage pain during LI for TOP.

The primary aim of our study was to measure the analgesic efficacy of the SSTS during TOP and assess patient satisfaction in nulliparous and multiparous women. The secondary aims were to report the maternal outcomes after the STSS administration and determine the need for rescue therapy.

## Methods

We conducted an observational, retrospective, single-center study to describe the clinical profile of the STSS for analgesia in nulliparous and multiparous women who underwent LI for TOP. The study was designed in accordance with the Strengthening the Reporting of Observational Studies in Epidemiology guidelines [20]. The study protocol was reviewed and approved by the local Ethics Committee of ASST Papa Giovanni (REG. SPERIM. N. 185/21).

Pregnant women undergoing LI for TOP were consecutively enrolled between January 2020 and May 2021 from the obstetric department of a tertiary referral hospital in northern Italy (ASST Papa Giovanni XXIII; Bergamo). Written informed consent was obtained from all patients for the use of the STSS and the processing of personal or clinical data. The following individuals were excluded: (1) those aged < 18 years, (2) those in their first trimester of pregnancy, (3) those with a history of opioid addiction or allergy, and (4) those with cognitive or psychiatric disorders.

The primary aim of our study was to measure the analgesic efficacy of the SSTS during TOP and to assess patient satisfaction in nulliparous and multiparous women. The secondary aims were to report the type and rate of the STSS-related side effects, evaluate adverse events, and determine the need for rescue therapy.

LI was initiated by a gynecologist using gemeprost, misoprostol, or dinoprostone following the obstetric protocol of the institution. In the case of prolabor rupture of membranes, no induction was necessary. Detailed information on the inductive protocols are illustrated in Supplementary Fig. 1.

After enrollment, the anesthesiologist provided patient education on pain assessment, employing a numerical rating scale (NRS) for pain (11-point scale ranging from 0, indicating no pain, to 10, denoting the worst imaginable pain), and instructions on how to use the self-administration device to administer one tablet of sufentanil whenever the pain level exceeded 3 on the NRS. If the NRS score was > 3, 1 g of additional acetaminophen was administered every 6 h, despite the use of the sufentanil as a rescue drug. Ondansetron (4 mg) was prescribed for nausea or vomiting.

Patient satisfaction was evaluated using a 5-point rating scale (1 = not satisfied, 2 = poorly satisfied, 3 = satisfied, 4 = highly satisfied, 5 = completely satisfied).

Demographic data and clinical characteristics (including age, body mass index, maternal parity, and gestational age), reason for LI, pain severity, number of tablets of sufentanil required, total and hourly doses of sufentanil, duration of SSTS use, patient satisfaction, side effects (such as nausea or vomiting, itching, sedation, and migraine), adverse effects (such as respiratory failure, severe arrhythmias, or coma), and the need for additional analgesics were recorded.

Sedation was evaluated using University of Michigan Sedation Scale (0 = awake and alert; 1 = minimally sedated: tired/sleepy, appropriate response to verbal conversation, and/or sound; 2 = moderately sedated: somnolent/sleeping, easily aroused with light tactile stimulation or a simple verbal command; 3 = deeply sedated: deep sleep, aroused only with significant physical stimulation; 4 = unarousable).

### Statistical analysis

Statistical analyses were performed using STATA 17.0 BE software (Basic Edition; College Station, TX, USA)*.* Categorical data were expressed as absolute and relative frequencies. If normally distributed, numerical data were expressed as the mean ± standard deviation; if non-normally distributed, they were presented as the median [interquartile range]. The normality of the distribution was assessed using the Shapiro–Wilk test. Categorical data were compared using the chi-squared test or Fisher’s exact test, as appropriate. Continuous variables were compared between the two groups using Student’s *t*-test for unpaired data or the Wilcoxon rank-sum test, as appropriate. Two-way scatter diagrams and Spearman’s correlation coefficients were used to assess the relationships between numerical variables. Statistical significance was set at *P* < 0.05.

Based on clinical observations and available data during the study planning phase, we estimated a mean difference in the sufentanil dose of about 50 mcg with a standard deviation of 60 mcg between nulliparous and multiparous women. Under these assumptions, considering an α error of 0.05 and a power of 0.80, we planned to recruit at least 48 patients, with 24 patients allocated per group.

## Results

Between January 2020 and May 2021, 50 patients were admitted to our ward for LI for TOP. Of these, two were excluded because they did not meet the inclusion criteria. Finally, 48 women were enrolled (27 women for medical abortion and 21 for intrauterine fetal death). The demographic and clinical characteristics of the study population are summarized in Table 1.

**Table 1:** Demographic data and the clinical characteristics of the study population

	***Patients (n* = *48)***
Age, years	34.9 ± 4,3
Weight, kg	64.9 ± 7
Height, m	1.65 ± 0.04
BMI, kg/m^2^	23.7 ± 2.4
Parity, *n* (%) Nulliparous Multiparous	24 (50) 24 (50)
Causes of TOP, *n* (%) IUFD MA	21 (43.7) 27 (56.3)
Gestational age, weeks	17 [15–20]

Data are presented as absolute and relative frequencies, mean ± standard deviation, and median [interquartile range]

*BMI* body mass index, *TOP* termination of pregnancy, *IUFD* intrauterine fetal death, *MA* medical abortion

**Fig. 1:**
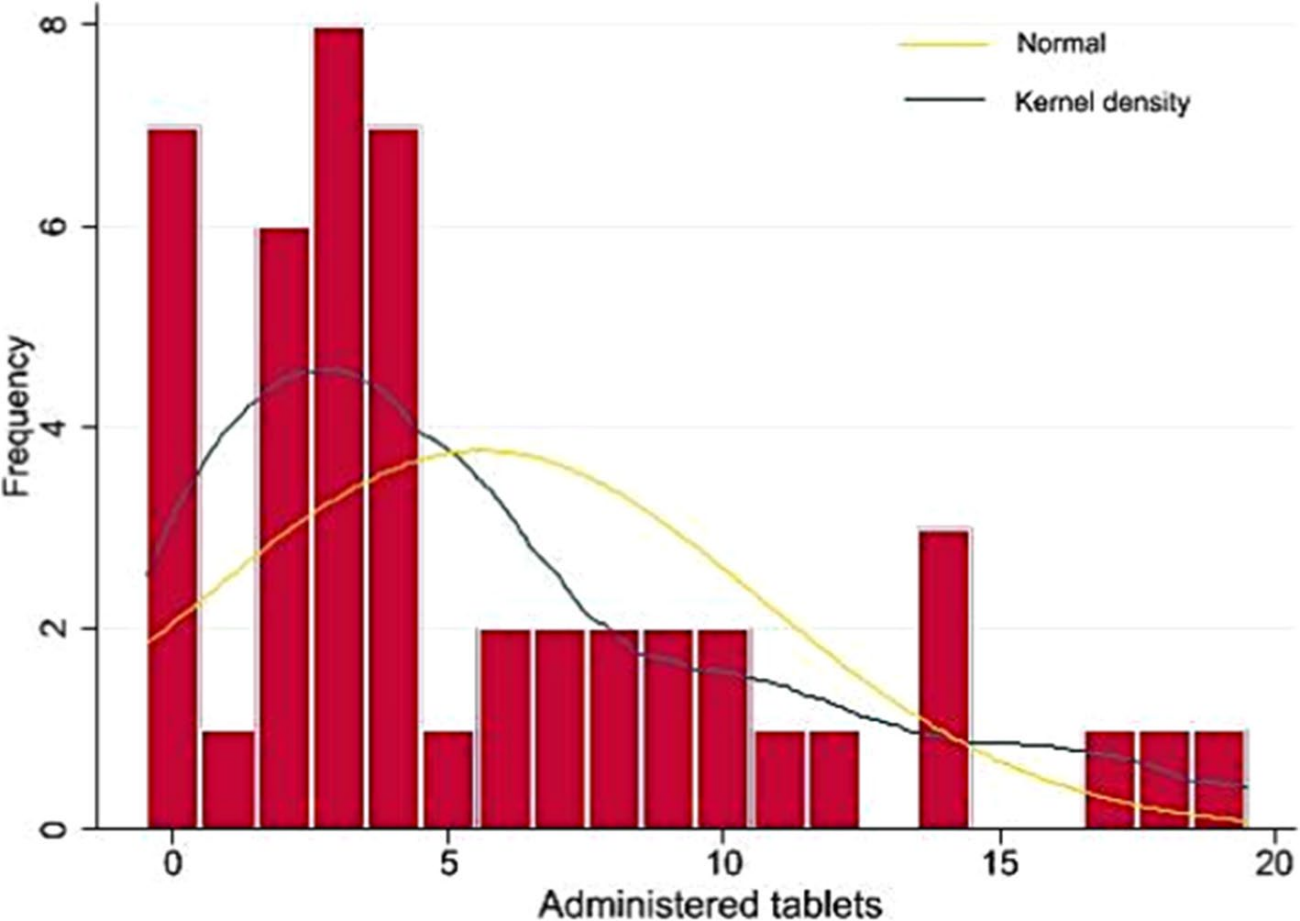
Histogram of the administered tablets. “Frequency” refers to the number of patients, and “administered tablets” refers to the number of tablets consumed

The median drug administration time (time between the first tablet taken and delivery) in the entire cohort was 296.5 [65–1060] min, the median total dose of the drug administered was 60 [30–127.5] mcg, and the median number of doses administered was 4 [2–8.5] tablets (Fig. 1). The median hourly dose of administered drugs was 12.7 [3.424.4] mcg/h.

**Fig. 2:**
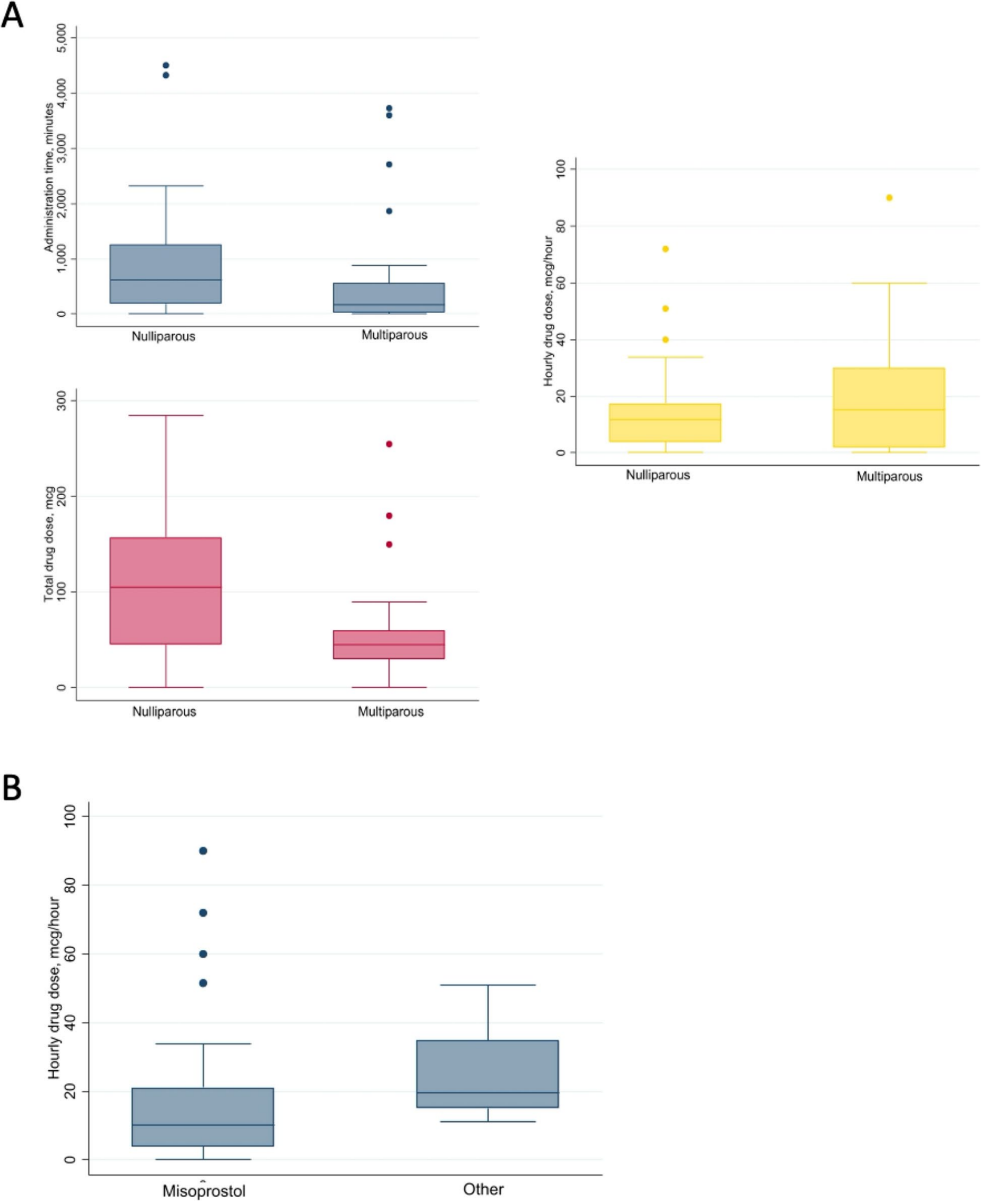
**A** Comparison of time of administration, total drug dose, and hourly drug dose in nulliparous versus multiparous women. **B** Comparison of hourly drug dose between patients induced with misoprostol and those using other protocols

In our study population, nulliparous women required a significantly higher dose of the drug than did multiparous women (105 [45–57.5] mcg vs. 45 [30–60] mcg, *P* = 0.01). The administration time was also found to be significantly longer in nulliparous women compared with multiparous women, with respective median durations of 625 [187–1253] min and 165 [30–562] min (*P* = 0.05). No significant difference in the hourly drug dose was observed between nulliparous and multiparous women, with respective median doses of 11.6 [3.8 to 17.5) mcg/h and 15.2 [1.9 to 30] mcg/h (*P* = 0.70) (Fig. 2A).

Comparing the different labor induction protocols, patients who received misoprostol required a significantly lower hourly drug dose as compared to the others (10 [3.8–21] mcg/h vs 19.4 [15–34.8] mcg/h, *P* = 0.03) (Fig. 2B). The labor duration was longer in patients who received misoprostol, although not statistically significant (300 [30–1260] min vs 180 [90–440] min, *P* = 0.39). We did not observe any significant difference between the two groups in the total dose of drug administered (*P* = 0.50).

Moreover, we observed a significant direct correlation between the number of tablets self-administered and the duration of labor (Spearman’rho = 0.661, 95% confidence interval [CI]0.463–0.795, *p* < 0.01) but an inverse correlation between the hourly drug dose and the duration of labor (Spearman’s rho =  − 0.883, 95% *CI* [− 0.936 to − 0.791], *P* < 0.01).

Regarding satisfaction, 40 patients (83.4%) were satisfied or completely satisfied, whereas only 8 patients (16.6%) reported dissatisfaction with the SSTS analgesic protocol. Among all patients who reported being “poorly satisfied” or “not satisfied,” the reasons were attributed to side effects in four patients, difficulty in using the SSTS device in two patients, induction lasting longer than 72 h in one patient, and false expectations from the device in one patient.

We observed a significant association between parity and the level of satisfaction, with multiparous women being more satisfied than were nulliparous women (*P* = 0.03) (Table 2). Conversely, there were no significant associations between the reported side effects and parity (*P* = 0.27) (Table 3).

**Table 2:** Rate of satisfaction in the entire cohort and the association between parity and the level of satisfaction

	***All patients (n* = *48)***	***Nulliparous women (n* = *24)***	***Multiparous women (n* = *24)***	p-value*
**Level of satisfaction, *n* (%)**				**0.03**
1 — not satisfied	3 (6.2)	3 (12.5)	0 (0)	
2 — poorly satisfied	5 (10.4)	3 (12.5)	2 (8.4)	
3 — satisfied	16 (33.4)	11 (45.8)	5 (20.8)	
4 — highly satisfied	18 (37.5)	6 (25)	12 (50)	
5 — completely satisfied	6 (12.5)	1 (4.2)	5 (20.8)	

Data are presented as absolute and relative frequencies

*Fisher’s exact test

**Table 3:** Frequencies of side effects in the entire cohort of patients

	***All patients (n* = *48)***	***Nulliparous women (n* = *24)***	***Multiparous women (n* = *24)***	p-value*
***Side effects, n (%)***				**0.27**
*None*	27 (56.2)	11 (45.8)	16 (66.7)	
*Nausea/vomiting*	15 (31.2)	10 (41.7)	5 (20.8)	
*Itching*	1 (2.1)	1 (4.2)	0 (0)	
*Sedation (UMSS^)*	4 (8.3) (1 = minimally sedated)	2 (8.3) (1 = minimally sedated)	2 (8.3) (1 = minimally sedated)	
*Migraine*	1 (2.1)	0 (0)	1 (4.2)	
*Discontinuation of SSTS°*	1 (2.1)	1 (4.2)	0 (0)	**1.00**

Data are presented as absolute and relative frequencies. The total percentage is more than 100% as patients exhibited more than one side effect

*Fisher’s exact test

*UMSS*^ University of Michigan Sedation Scale, *SSTS°* sufentanil sublingual tablet system

None of the patients experienced severe adverse effects. The most frequently reported side effects were nausea and vomiting, which occurred in 31.2% of patients. Notably, nausea and vomiting were the primary causes of discontinuation of STSS in only one patient (2.1%). The frequencies of side effects across the entire patient cohort are detailed in Table 3.

In our sample, only four patients (12%) required the administration of acetaminophen due to insufficient analgesia, and in one patient (2.1%), we changed the analgesic protocol, switching to intravenous (IV) morphine for device blockade.

Regarding the sufentanil dosage administered, we decided to compare the usage of the analgesic drug with the gestational age of women who underwent TOP.

**Fig. 3:**
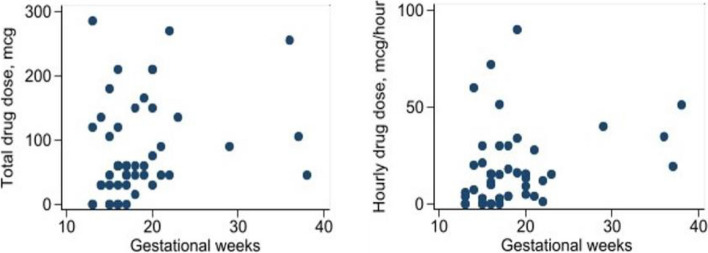
Correlation of gestational weeks with the total dose and hourly dose of the administered drug

The correlation analysis revealed a direct correlation between gestational weeks and the total dose of the drug administered (Spearman’s rho = 0.315, 95% *CI* [0.035–0.577], *P* = 0.03), as well as with the hourly dose of the drug administered (Spearman’s rho = 0.349, 95% *CI* [0.073–0.577], *P* = 0.01) (Fig. 3). When evaluating gestational weeks and the total drug administration time expressed in minutes, no significant correlation was found (Spearman’s rho = 0.099, 95% *CI* [− 0.190–0.373], *P* = 0.10).

## Discussion

Labor pain is a unique human experience that differs among women in terms of intensity and duration. Pain can be worsened by emotional stress, as observed in TOP. Achieving adequate pain control is essential but challenging.

The primary aim of our study was to measure the analgesic efficacy of the SSTS by comparing nulliparous and multiparous women, as parity influences pain severity [5–8]. Sublingual sufentanil was effective in both nulliparous and multiparous women with minimal side effects.

To better evaluate and compare the severity of pain, we analyzed indirect parameters, such as the total and hourly doses, because the duration of labor differs among patients. In our sample, nulliparous patients required more sufentanil tablets than do multiparous patients; however, there was no significant difference in the hourly dose (Fig. 2A). Nulliparous individuals generally have longer induction and labor times than multiparous individuals do, which justify the increased use of tablets. The use of analgesic medication and pain were significantly correlated with increased doses of prostaglandin analogs [21].

In contrast to findings from the existing literature, we were able to determine the hourly dosage using the SSTS and discovered not only that pain is likely to have the same intensity in both nulliparous and multiparous women but also an inverse correlation between hourly dose and duration of labor. Labor was more painful when the body responded faster to induction and cervical dilatation.

When comparing the different labor induction protocols, it was observed that patients who received misoprostol required a significantly lower hourly drug dose and had a longer labor length (Fig. 2B) [22].

The gestational age also affected the degree of pain. Gestational age was significantly related to both the total and hourly doses of sufentanil required by the women (Fig. 3), confirming that gestational age is a predictor of severe pain and higher drug use during labor for TOP [5–7, 23, 24].

In our study, we recorded a broad spectrum of sufentanil consumption (Fig. 1). Notably, 7 patients did not require any tablets, whereas the others required between 17 and 19 tablets. One explanation for this result is the heterogeneity of patients undergoing TOP in terms of parity, gestational age, and cause of TOP (medical abortion or intrauterine fetal death). This emphasizes the importance of patient-controlled analgesia [12] and suggests that analgesic prophylaxis may be unnecessary [21].

In the literature, a high proportion of patients (20 to > 80%) require rescue therapy employing various analgesic protocols to control pain during LI for TOP [8, 11]. In our study, only four patients (12%) required the administration of acetaminophen owing to insufficient analgesia. Considering the efficacy of NSAIDs, acetaminophen, IV opioids, or other medications during LI for TOP reported in other studies [8, 11], our results show a higher efficacy of the SSTS.

The majority (83.4%) of women using the SSTS were satisfied (Table 2), and 50% of the patients reported high or full satisfaction, regardless of the side effects or the need for a rescue dose. Multiparous participants exhibited significantly higher satisfaction levels, compared with nulliparous participants, probably because the latter group experienced longer labor. Only eight women reported being not satisfied, primarily citing reasons such as side effects, false expectations, or the length of labor induction. It is of great relevance to educate patients about the SSTS to reduce false expectations and promote the correct use of the device. Moreover, the SSTS was preprogrammed to dispense sufentanil for up to 72 h.

In one patient whose LI lasted over 72 h, we replaced the SSTS with a continuous IV infusion of morphine PCA, which was initiated and monitored by the clinicians. The STSS was well-tolerated by the patients and improved hospital admission by allowing mobility without the limitations of IV tubing or PCA infusion pumps. The SSTS reduces the risk of analgesic gaps and increases comfort and adherence to treatment [25].

In our study, we did not observe severe adverse effects, such as respiratory failure, severe arrhythmias, or coma. The most frequent side effects reported were nausea and vomiting (Table 3), which were well-controlled by antiemetic medications. Nausea and vomiting are common side effects of opioids and have been consistently reported as the most frequent side effects in previous studies where opioids were administered [16, 19]. Parity did not influence the incidence of adverse effects (Table 3). A small percentage of the patients (8.3%) reported minimal sedation (Table 3). Some patients reported minimal sedation with sufentanil as a positive experience in the TOP setting because it allowed for rest and anxiety relief. Lang et al. found that anxiety during labor is a predictor of pain [26]. Anxiety can stimulate the sympathetic nervous system and release stress hormones such as noradrenaline, cortisol, and adrenaline, increasing the severity and duration of labor [27]. Therefore, minimal sedation associated with efficient pain reduction induced by the SSTS may be considered a positive side effect, as it helps minimize the psychological distress associated with LI in TOP.

### Novelties and strengths of the study

Sufentanil is routinely administered to control acute postsurgical pain or during labor in conjunction with epidural analgesia or anesthesia. Sublingual sufentanil is effective for pain management in TOP and is less invasive, compared with epidural and intravenous administration of other opiates. Furthermore, our study aimed to improve analgesic control during the stressful period of TOP. To date, international guidelines have not provided an effective analgesic strategy to control this type of pain [9–11].

### Limitations of the study

Nevertheless, our study has some limitations. This was a preliminary assessment of the effectiveness of the SSTS; however, we did not compare it with other analgesic protocols, such as PCA with morphine or scheduled administration of NSAIDs/acetaminophen. Further, randomized studies are required to compare self-administered sublingual sufentanil with other PCA protocols.

In addition, the pharmaceutical company terminated the license supply agreement for the SSTS. The decision to discontinue the SSTS was not motivated by clinical, safety, or efficacy reasons. However, a new sublingual sufentanil formulation has recently been approved and is currently available [28]. Our positive preliminary results and the analgesic strategy described in this study could be adopted in the future for the management of LI in TOP.

## Conclusions

This study presented the first data on the application of sublingual sufentanil, which has been shown to be effective in treating pain during induced TOP in nulliparous and multiparous women. Sublingual sufentanil was well-tolerated and had a favorable side-effect profile. An important advantage of the SSTS is the possibility for women to determine when to take the drug. The ability to manage pain independently is crucial, even from a psychological perspective, to guarantee that women maintain agency over their primary role in the process.

### Supplementary Information

Additional file 1: Figure 1. The flow diagram shows the enrolment process and inductive protocols. MA* = medical abortion, IUFD^ = intrauterine fetal death, PROM = prolabor rupture of membranes.

## Data Availability

Data supporting the findings of this study are available upon request from the corresponding author (B. M.). The data are not publicly available because of privacy and ethical restrictions.

## Abbreviations

LI: Labor induction
TOP: Termination of pregnancy
NSAIDs: Nonsteroidal anti-inflammatory drugs
PCA: Patient-controlled analgesia
SSTS: Sufentanil sublingual tablet system
NRS: Numerical Rating Scale
BMI: Body mass index
IUFD: Intrauterine fetal death
MA: Medical abortion
