# Supplementary material for: Sublingual sufentanil for patient-controlled analgesia during labor induction for pregnancy termination: an effective and well-tolerated approach
Source: J Anesth Analg Crit Care. 2024 Jul 8;4:41. doi: 10.1186/s44158-024-00177-z (PMC11229235; doi:10.1186/s44158-024-00177-z)
Supplement: Supplementary file 1 — Additional file 1: Figure 1. The flow diagram shows the enrolment process and inductive protocols. MA* = medical abortion, IUFD^ = intrauterine fetal death, PROM = prolabor rupture of membranes. [file 44158_2024_177_MOESM1_ESM.docx]

**Supplementary Material**

***Figure 1.*** *The flow diagram shows the enrolment process and inductive protocols.*

21 IUFD^

Labor induction protocol

- Protocol I: 3 patients
- Protocol II: 24 patients
- PROM: 1 patient

Labor induction protocol

- Protocol I: 3 patients
- Protocol II: 14 patients
- Protocol III: 3 patients
- PROM°: 1 patient

27 MA*

1 excluded: age < 18 years

1 excluded: trimester I

48 included

50 pregnant women undergoing TOP

Protocol I: gemeprost 1 ovule of 1 mg every 3 h, up to a maximum of 5 ovules.

Protocol II: misoprostol 400 mg every 3 h, up to a maximum of 5 doses. In case of failed induction mifegyne 600 mg, and after 24 h, another cycle of misoprostol was provided.

Protocol III: dinoprostone 10 mg

*MA* = medical abortion, IUFD^ = intrauterine fetal death, PROM° = prolabor rupture of membranes*
